# A Soft Haptic Glove Actuated with Shape Memory Alloy and Flexible Stretch Sensors

**DOI:** 10.3390/s21165278

**Published:** 2021-08-04

**Authors:** Silvia Terrile, Jesus Miguelañez, Antonio Barrientos

**Affiliations:** Centre for Automation and Robotics (CAR), Universidad Politécnica de Madrid—Consejo Superior de Investigaciones Científicas, 28006 Madrid, Spain; jesus.perez.miguelanez@alumnos.upm.es (J.M.); antonio.barrientos@upm.es (A.B.)

**Keywords:** haptic device, shape memory alloy, flexible stretch sensor

## Abstract

Haptic technology allows us to experience tactile and force sensations without the need to expose ourselves to specific environments. It also allows a more immersive experience with virtual reality devices. This paper presents the development of a soft haptic glove for kinesthetic perception. It is lightweight and soft to allow for a more natural hand movement. This prototype actuates two fingers with two shape memory alloy (SMA) springs. Finite element (FE) simulations of the spring have been carried out to set the dimensions of the actuators. Flexible stretch sensors provide feedback to the system to calculate the tension of the cables attached to the fingers. The control can generate several recognizable levels of force for any hand position since the objects to be picked up can vary in weight and dimension. The glove can generate three levels of force (100, 200 and 300 g) to evaluate more easily the proper functioning. We realized tests on 15 volunteers simulating forces in various order after a quick training. We also asked volunteers about the experience for comfort, global experience and simplicity). Results were satisfactory in both aspects: the glove fulfilled its function, and the users were comfortable with it.

## 1. Introduction

Human beings depend on five senses, and haptic technology focuses on touch. It arose because of the amount of information we can perceive through touch. Information can be added to what we already perceive with other senses thanks to screens or other similar devices. However, haptics are especially interesting in medicine for delicate surgery or rehabilitation [1] or for handling highly unstable and radioactive elements to avoid exposure to certain risks. However, it also finds important development in leisure activities, such as video games, to provide an increasingly immersive experience. Kinesthetic haptic gloves allow the simulation of the grip of objects in different ways, for example, by preventing the advancement of the fingers or by performing an extension movement.

Currently, a variety of haptic glove prototypes and commercial models exist that create different sensations (touch, grip, heat), but this research will focus only on devices that perform the grip sensation.

The first way to classify haptic gloves is to distinguish between desktop and wearable prototypes. Desktop devices have the great advantage of high power, and they realize the force necessary to prevent hand movements through the motors located at their base and several transmissions. However, the joint structure generates high rigidity. In addition, they require space, and limit the range of motion available. Cyberforce [2], from the company CyberGlove Systems, is one of the desktop gloves with actuators on the back of the hand. Movement limitation could be seen as an advantage if the aim is to limit the advance of the hand. The HIRO III [3] also belongs to this category although the actuators are at the fingertips. Other lesser-known examples are SPIDAR-MF [4] or HGlove [5].

In recent years, however, more devices of the wearable type have been developed. Many models use pneumatics despite the complexity and large volume that this type of actuation requires. Increasing the number of DOF (Degrees of Freedom) presented by the glove implies a new, greater volume than the alternatives. In addition, the compressor and the noise it generates have to be taken into account. The Jamming Tubes [6] prototype simulates the grip of not-heavy objects using the principle of jamming. It is quite a bulky system for two-finger control. Air bladders in the palm and on the back, depending on the desired sensation, are widely used. A recent pneumatic model is the Haptx Glove [7], which has several small bladders on both the palm and the fingertips. These are inflated independently to obtain a faithful tactile simulation that allows differentiating in the same fingertip depending on the contact location. In addition, these gloves have several motors for grip sensation.

The other most widely used actuators are DC motors in tendon-driven models, such as Hand Exoskeleton [8]. They can precisely control the range of movement with the help of an encoder. However, it is a bulky and not lightweight design. The size will be more accentuated with the increase in the DOF since they are associated with an increase in the number of DC motors. Similarly, the more force required, the more powerful the motors. Therefore, for some force ranges, motors end up being outside the glove. The TeslaSuit company [9] presented in early 2020 a haptic glove with servo motors.

In the case of the model called MRAGES [10], a magnetorheological fluid (MRF) was used inside a piston. A magnetic field was varied to control the movement and, consequently, fluid viscosity. Thus, high forces can be achieved with light equipment and safety. In this case the actuators provided forces up to 6 N; but with simple changes in design can create much higher forces. However, these types of fluids have a very high cost and a slow reaction time.

Another alternative is the use of a Shape Memory Alloy (SMA). When an SMA is heated, it recovers the shape previously memorized in heat treatment, so its length can be affected and used to restrict movement. The ExoPhalanx [11] is a glove that takes advantage of the SMA’s ability to compress as a brake for the cable, making it more complicated to move around. The main disadvantage of this design is the all or nothing control over the force. In this way, the weight grasped in the simulation cannot be controlled and depends on the strength exerted by the fingers. The only thing that can be characterized in the simulator would be the size of the object. In the case of the prototype [12], the intermediate areas of each phalanx are compressed by SMA rings that contract when current is passed through them, causing the sensation of pressure. However, the advancement of the fingers is not impeded, so the grip it realizes is not realistic. In addition, as soon as several consecutive fingers are implemented in the glove, the rings can rub and reduce the naturalness of the movement.

A market model, widely accepted for its lightness and power, is the DextrES [13] glove. With a high voltage (2000 V) and very low amperage (500 µA maximum), it offers forces of up to 20 N. It uses two metal bands for each finger, one fixed on the wrist and the other located along the finger. As it moves, its band slides over the one on the wrist. When it is necessary to retain the movement, the current circulates through the metal bands, and by the electrostatic effect, the bands adhere, preventing movement. However, a costly source is necessary to get the voltage and amperage values previously mentioned.

Finally, to recreate the grip, and therefore experience a force that opposes the penetrating object, some prototypes [14] take advantage of the force generated by the user’s opposing fingers by using elastomeric dielectric actuators. Depending on the size of the grasped object, the actuators will lock in different positions. The main disadvantages are the inability to recreate actions that involve a single finger, or the size grasp limited by the possibility of the compression of these actuators.

The most widely used models are those with pneumatic actuators and DC motors, whether desktop or wearable. Although the other actuators (MRF, SMA) are less used, they offer essential advantages when designing a soft haptic glove that is lightweight and flexible but at the same time powerful. In [15], an updated state-of-the-art wearable haptic devices is available.

This work presents the development of a soft haptic glove actuated with SMA springs. It is able to generate a kinesthetic perception in the user. Usually, SMA actuators are used in an “all or nothing” manner, but in our work, we achieve three different actuation levels to simulate three different sensations (three different forces). Our results have the purpose of showing how SMA actuators could be an interesting option for haptic devices. First, the development of the prototype is illustrated in Section 2, explaining all the design decisions and the components. Then, in Section 3, the actuators are analyzed deeply through finite element (FE) analysis. Next, Section 4 illustrates the control and characterization of the system. Then, experiments and results are discussed in Section 5, followed by the conclusions in Section 6.

## 2. Soft Glove Prototype

The requirements for the design of the glove were: soft material, soft actuators with a smooth weight/power ratio, lightweight, wearability, and portability. Softness is essential in this device since the stiffness would not have allowed the realistic simulation of lighter objects.

**Figure 1:**
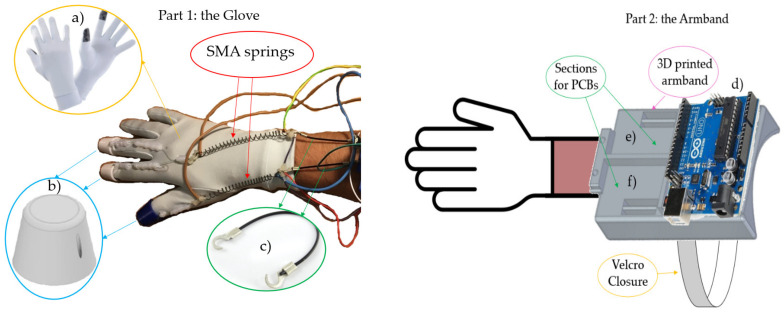
The two principal parts of the haptic glove. (**a**) Glove made of elastic fabric. (**b**) 3D printed finger cap (**c**) Flexible stretch sensors (**d**) Arduino UNO (**e**) electronic circuit for SMA control (**f**) electronic circuit for stretch sensors.

The device consists of two principal parts: (1) a glove with actuators and cables, (2) an armband with electronics and power cable. A flexible stretch sensor (Images Scientific Instruments) connects the two parts. The selected glove is made of elastic fabric (polyester and elastane) that provides elasticity and thermal insulation (Figure 1a).

A nylon thread of 0.5 mm diameter connects the fingertips to the actuators. This material is lightweight, flexible, and robust. The nylon thread passes through a Teflon™ pipe to avoid friction with the glove and realizes a smoother movement. It presents an outer diameter of 4 mm and an inner diameter of 2 mm, and it is glued to the glove with a hot glue gun. This solution was more straightforward than the option of sewing tubes due to the fabric glove. The pipe is divided into two parts to guide the thread but does not interfere with the finger’s movement.

The nylon thread is not directly connected to the glove because this could possibly tear the fabric through use. Therefore, a three-finger cap have been designed and printed in 3D with PLA. They are joined to the glove as the Teflon pipe and present a small hole to realize the connection with the thread.

On the other end, the cable is connected to the SMA actuator. This connection is challenging because a higher temperature reaches SMA during this function. Therefore, to avoid fusion of the nylon (its diameter is tiny), thermal insulation with two plastic clamps is necessary.

The same insulated connection is used between the SMA and the flexible stretch sensor. In this second case, the insulation is even more important for avoiding incorrect measurements that could happen without it. Connected to the SMAs are two flexible stretch sensors.

The manufacture of the glove was very simple. The only bespoke parts were the 3D printed finger caps and the armband (and, of course, the electronics). All other components were purchased (glove, springs, sensors).

These sensors offer reasonably precise measurements of the tension state of the cables. The total length, including the two hooks, is 5 cm. When the sensor is relaxed, the rubber has a nominal resistance of approximately 200 ohms per centimeter. According to the manufacturer, the resistance gradually increases with the sensor stretching up to 150% of its original length (5 cm × 150% = 7.5 cm).

By establishing a correct relationship between electrical resistance and deformation, we indirectly obtained a measurement of the tension in the sensor as long as we are in the linear zone of deformation. In Section 4, the procedure for obtaining these measurements will be described in detail. The elastic property of the sensor will also facilitate the recovery of the initial state of the SMA since it will pull it back to its nominal length. Cooling is the most time-consuming phase in the SMA cycle. However, as explained in [16], placing a bias spring with a fixed-grip on one side of the SMA favors the recovery of the spring since, as it cools and gives way, the system will return to its initial disposition (or at least close to it).

**Figure 2:**
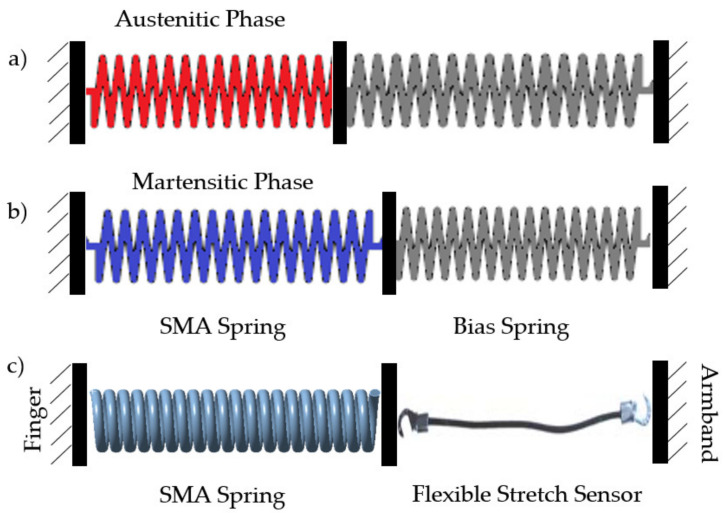
SMA-spring actuators (**a**,**b**) The idea as illustrated in [16] (**c**) the idea as implemented in our work.

**Figure 3:**
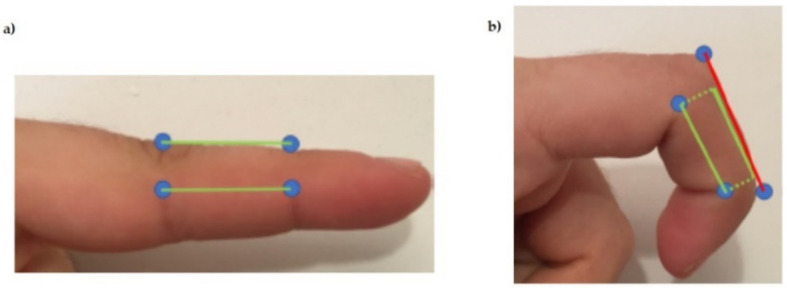
(**a**) Finger fully stretched (minimum distance between upper joint area). (**b**) Bent finger (maximum distance between the upper part of the joints).

In the present work, we changed the springs with elastic sensors (as shown in Figure 2c) to provide information in addition to fulfilling this recovery function. Moreover, this design solved one of the problems of haptic glove designs with cables that pass in the upper area of the fingers: the turning radii and the need for it to have two fixed points at both ends. If the cable were to pass inside the fingers, as tendons do, this would not impede the total length required, which would always be constant. However, when these pass over the skin, some displacement at the cable ends because of the radii of gyration. This phenomenon happened when we actuated the SMA when the system was at rest. It gave rise to the appearance of a force that blocks the free movement of the hands. Figure 3 shows this effect between two joints which, if we add it to the one produced between the other finger and the wrist, will give rise to displacements that cannot be ignored.

We will therefore need some freedom at one end of the cable to move our fingers, and this can be achieved by deforming these sensors or the SMA. A high elasticity constant would block this freedom since we would need a high force for a movement that apparently should be without load. On the contrary, a constant that is too low to allow the fingers to move naturally would cause that variation in the length of the SMA that the spring must absorb to result in an inappreciable force, rendering the actuator useless. It makes the selection of sensors (used in this case also as springs) crucial.

The armband provided the electronics: it consists of a single part impressed with 3D printing (PLA) (Figure 1).

It is possible to identify three sections, each of which houses a specific circuit of the electronic system: the largest is for the Arduino UNO, and the other two are, respectively, for two PCBs (one for the SMA control and one for the stretch sensors reading). It also presents a grip zone for the sensors. This design allows the glove to be assembled and disassembled quickly. The face in contact with the arm is slightly curved to generate comfort for the user. A Velcro closure is used to fasten the armband. In this way, each user can adjust the size. In this case, the power supply is external. It reduces mobility but at the same time allows a more lightweight prototype. In the future, it will be possible to add a battery and realize a portable device.

**Figure 4:**
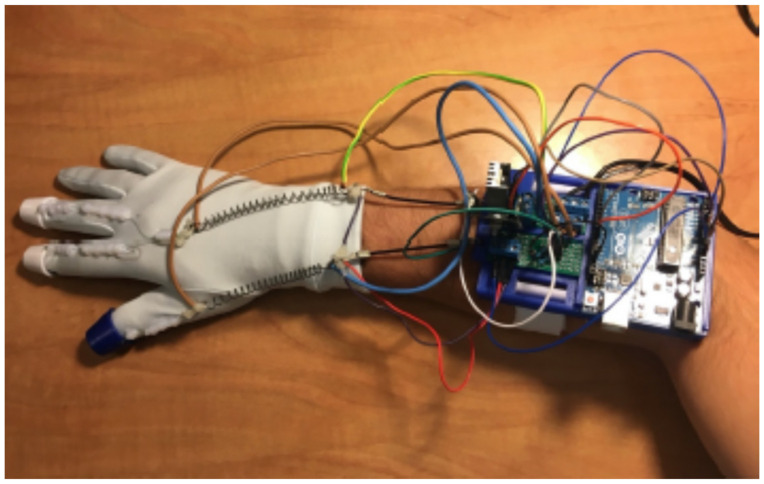
Final Haptic Glove.

To correctly use the device, the armband is placed closest to the elbow and, then the glove is put on. Finally, by stretching the springs as necessary, the sensors became anchored to the armband with some tension. All SMAs must be on the glove fabric since it acts as an insulator for the skin. The final prototype is shown in Figure 4.

## 3. SMA Springs: Characterization and Actuation

The actuators selected to realize this prototype were SMA springs for all the reasons explained in the state-of-the-art. Furthermore, different finite element simulations were realized with Ansys to choose the correct dimensions for the spring. Nitinol spring characteristics are shown in Table 1.

A first estimate of the spring force has been calculated with the Formula (1) [17]:

(1) $$F=\frac{\tau_{a\;}\;\pi d^{3}}{8D}\left[N\right]$$

The maximum force that the spring could carry out was about 12 N, being τ_a_ the maximum shear stress, d the wire diameter, and D the spring diameter. Unfortunately, the seller did not provide the mechanical properties of the material. So, the maximum shear stress (τ_a_ = 450 MPa) provided by another seller (in this case by the SAES getters group [18]) was used.

Finite element simulations were realized to validate this value. For the same reason as before, the values recommended by Ansys in their guide [19] have been used. They are shown in Table 2.

**Figure 5:**
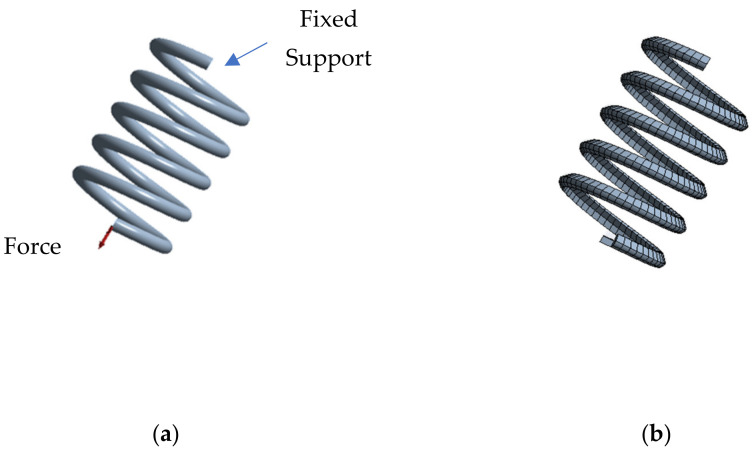
Model of the SMA spring (**a**) the geometrical model and boundary conditions, (**b**) finite element model (SOLID185 elements).

The force exerted by the spring does not depend directly on the number of turns, as shown in the formula (1). Therefore, the simulated spring (showed in Figure 5) presented a lower number of turns (5 instead of 21) to reduce the computational cost and simulation time. The spring had a fixed support at one end, while it presented a force that acted by stretching the spring at the other (Figure 5a).

**Figure 6:**
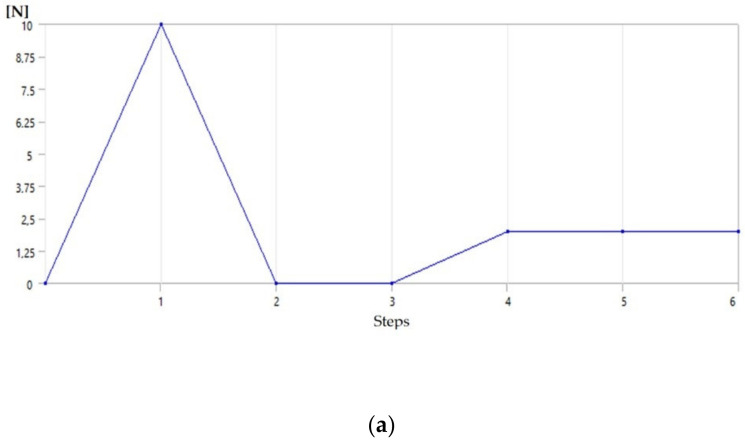

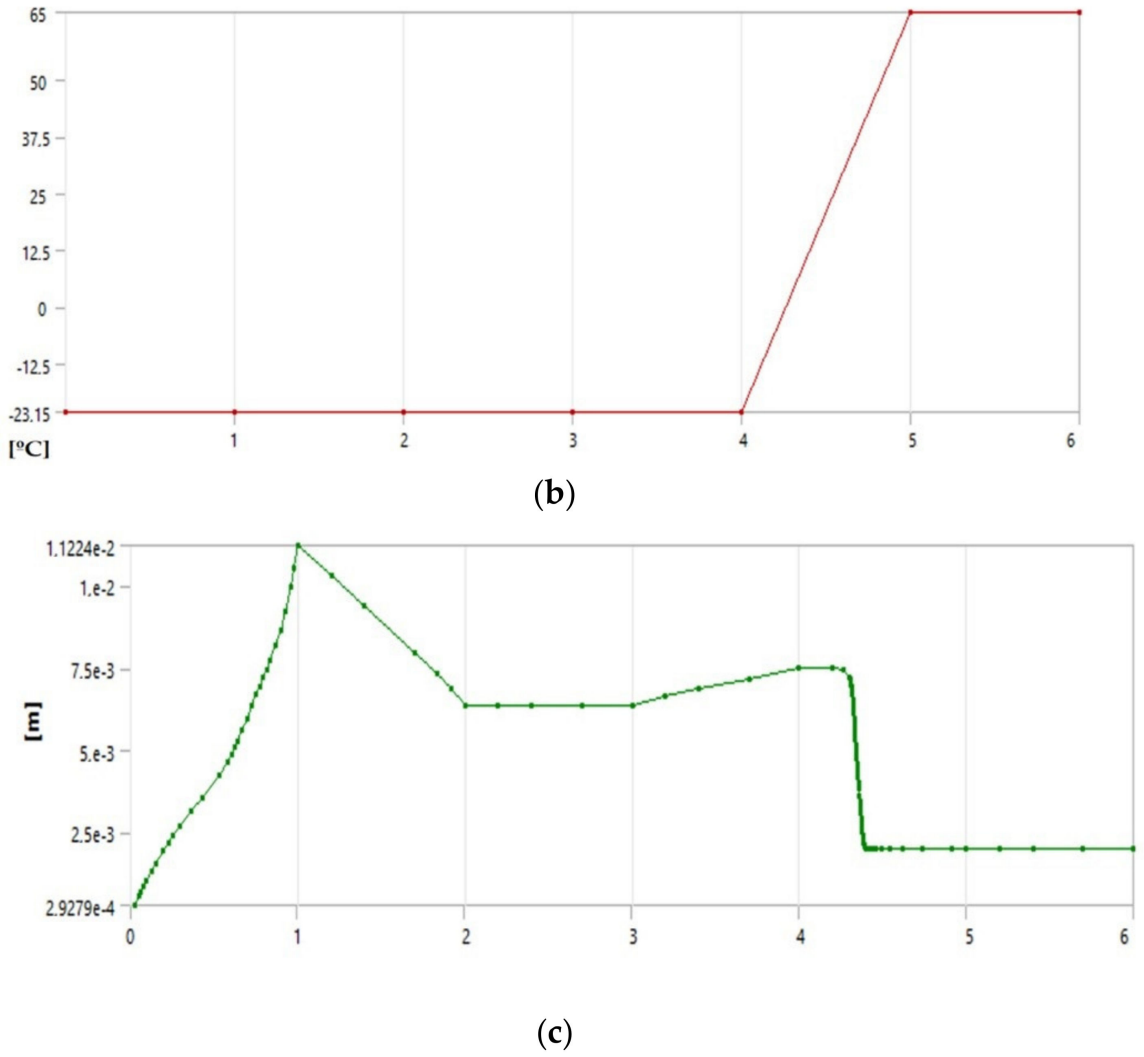
SMA spring analysis. (**a**) force applied for each step, (**b**) thermal conditions applied for each step, (**c**) total deformation for each step.

**Figure 7:**
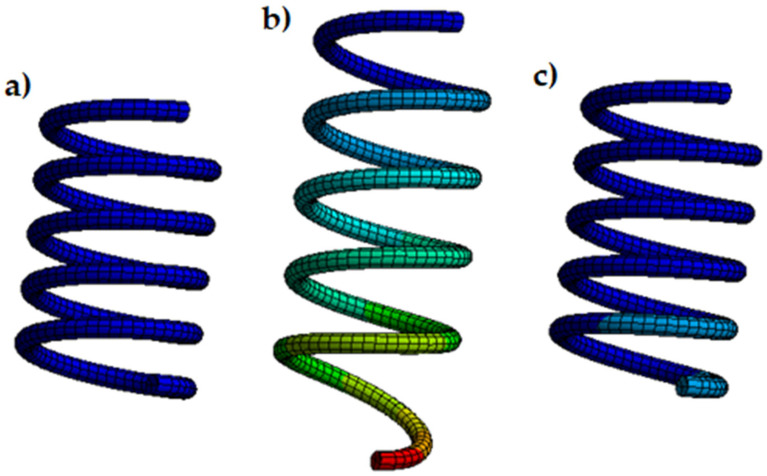
Deformed shape compared with undeformed shape. (**a**) spring at the initial step 0, (**b**) deformed spring after step 1, (**c**) the spring recovers its shape after the heating process.

The simulation consists of 4 main steps: (1) the spring is deformed by applying about 10 N of force at room temperature; (2) the force applied is removed and the spring does not recover its shape and maintains the plastic deformation; (3) no force or heat was applied to the spring, thus simulating its normal operating condition once mounted on the glove; and (4) the activation temperature provided by the seller was applied and, the spring fully recovers its shape, leaving zero initial deformation. Figure 6 shows the force and thermal conditions applied and the resultant deformation. Figure 7 shows how to change the spring shape during simulation.

After applying an initial force of 12 N, the spring does not fully recover its shape Therefore, the first estimate of the force was quite correct.

Performing several simulations, varying the initial load to which we subjected the spring, and therefore varying its initial deformation and keeping the thermal excitation the same, we observed that the contraction varied. This gave rise to variations in the resultant force as a function of the initial deformation, which will be verified experimentally later. Simulations also showed a higher deformation in the areas furthest from the anchor, as we will also see in the tests.

After the mechanical analysis, we proceeded to study the electrical circuit to actuate the springs. Their electrical resistance was verified experimentally to be equal to 1.3 ohms. The source used offered 12 V and up to 3 A, with a nominal power of 40 W. We selected the Mosfet model IRF530, channel *n*. With this model, we dissipated a maximum of 88 W.

Following the characteristic curve (data at 25 °C) offered by the manufacturer, for a gate-source voltage of 5 V (characteristic voltage of the Arduino pins), the drain current will be around 3 A. Therefore, we will not be able to act with duty cycles greater than 40–50% on the two SMAs simultaneously since the capacity of the source will be exceeded.

**Figure 8:**
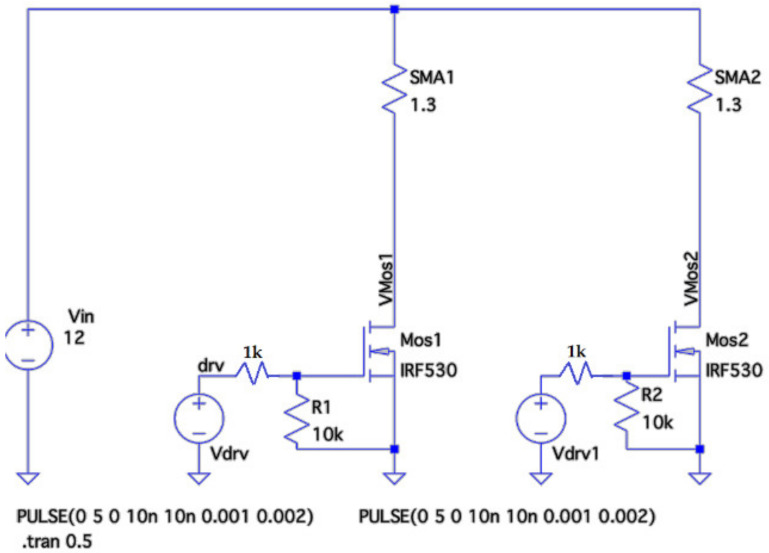
General schematic in LTspice.

The scheme used to control the Mosfet is that of Figure 8. The simulations have been carried out with LTspice.

To use these devices as haptic glove actuators, they have to exert different levels of force. Therefore, the SMA is usually used in an on/off manner, where the only possible states are when the austenitic transformation has been completed or not started. Analyzing any graph that shows the current-strain relationship for an SMA spring (for example, the one shown in Chapter 3 of the book in [20]), we saw that if we managed to position ourselves at intermediate points of the curves, we obtained different states of stress. Therefore, a perfect thermal balance had to be achieved. Depending on the dimensions of the chosen spring, it will be more or less complex. If, for example, it is small, it will heat up quickly with tiny variations in the current, making any intermediate point of the curve inaccessible. If we could access these areas of the curve, the main drawback became the hysteresis of the process. A priori, without a feedback system, we cannot know at which point of the curve diagram we are. One option would be to use thermal sensors that give us an idea of what percentage of the transformation has been completed, and what sense we are in (heating or cooling). However, this process seems quite complex since these two curves are not always the same, and they vary, as we have seen, with initial deformation.

The most straightforward alternative would be to ignore this hysteresis and find a point of equilibrium once the desired force was reached wherever we are on the curve.

We would act according to the force that being carried out at all times, by increasing the current if it is not enough or decreasing it if it were excessive.

We will, therefore, need a sensor capable of measuring this force.

## 4. Control

The control of the system was carried out with an Arduino UNO, a closed-loop control that gives feedback from the stretch sensor that provides the tension existing in the SMA-sensor assembly.

Once the tension state of the cables has been measured, the system performance varies. Therefore, the difference between the force required and the measurement is equal to 0, or, at least, close to it.

A PWM value that provides some stabilization in the actuator temperature at room temperature should be sought. It will not be completely accurate because, to remain stable in each situation, it will be necessary to supply different energy values depending on the desired force and different temperatures reached by the SMA.

In addition, it will depend not only on the needed force but also on other initial factors such as the initial deformation. It will also not be possible to ensure constant factors in each use. The error is defined as:

$$Error=F_{sensor}-F_{required}$$

When the force ($F_{sensor})$ is less than the demand ($F_{required})$, PWM values lower than stabilization will be necessary. If necessary, it could even correspond to a zero-duty cycle to protect the actuators. On the contrary, when the error is negative, we still have to contribute energy. In both cases, we observe that the necessary duty cycle will not exceed 4%. The PMW values depend on the calculated error, as will be explained better later, so the control is proportional.

To reach a constant force starting from rest, we cannot use a PWM of the order of those used to stabilize because heating the SMA to its transformation temperature would be on the order of minutes. For this reason, initially, the duty cycle will be 67% (this corresponds to a current value of 2 A). The spring will heat up in seconds. Once the desired force has been reached, the duty cycle must be reduced immediately to the mentioned values (less than 4%) to avoid complete austenitic transformation.

It is also possible to reduce the duty cycle before reaching the required force by taking advantage of the thermal inertia of nitinol.

In the beginning, when the value of the PWM is 0, the SMA should enter the warm-up phase. However, the system will always remain at rest until the force to simulate is not indicated.

Once the setup was finished, we read the analog ports (corresponding to the sensors) and calculate the tension in the cables. Next, we checked if the last reading from the serial port indicated a non-zero value for the force. If this were null, it returned to the beginning of the loop with the duty cycle equal to zero for both actuators.

**Figure 9:**
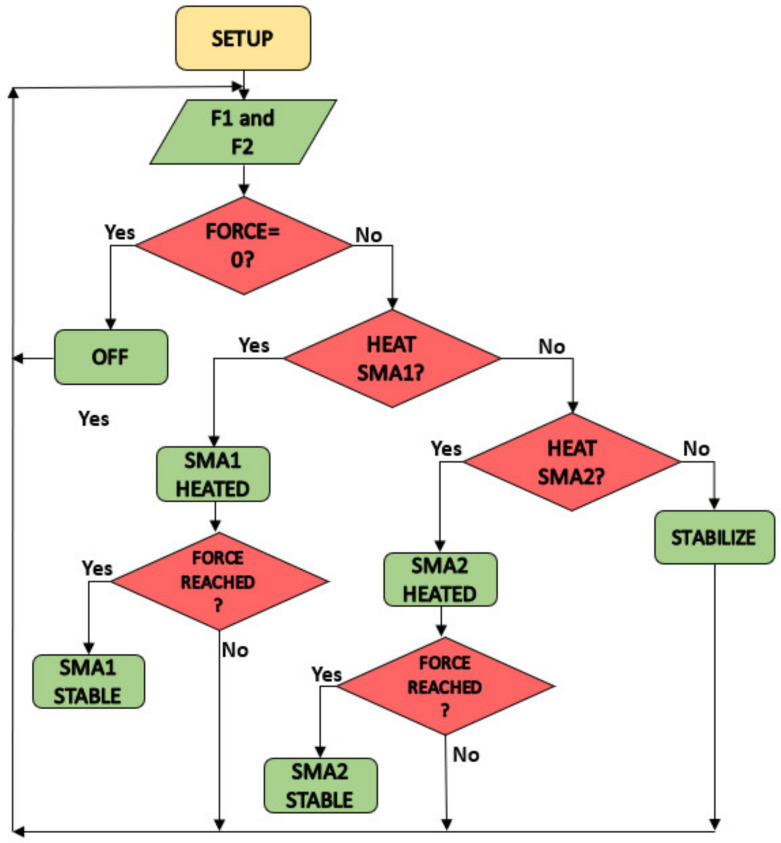
Code flow diagram in Arduino.

In the case of obtaining a correct value other than zero, the SMA will be heated. Once the SMAs reach the operating point, they will be assigned a PWM that stabilizes them. In Figure 9, the code flow is shown.

**Figure 10:**
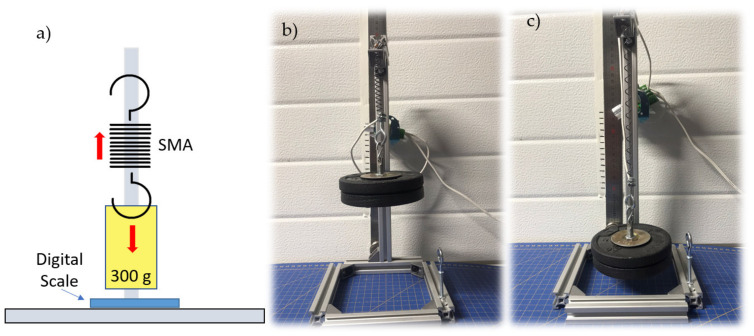
General setup for the experimental characterization of the SMA spring. (**a**) Scheme (**b**,**c**) Reality.

Several tests had to be carried out to realize this control as precisely as possible and achieve a reliable characterization of the nitinol springs and flexible sensors. The setup prepared to collect these data consisting of a structure that supports a bar from which a weight of 300 g hangs. It is greater than the 2 N required for each SMA. The SMA weight (3 g) is assumed to be negligible. Under the weight is a scale. Figure 10 shows the assembly previously described.

The procedure for obtaining the spring tension measurements was as follows:

1. The measure indicated by the scale was noted when there was still no current flowing.
2. The SMA was heated and how the measurement on the scale varies until it stabilized was observed.
3. The stabilized value was subtracted from the first one noted, and the tension experienced in the spring was obtained.

In the first tests, some parameters that may have conditioned the operation of the Nitinol actuator were varied, such as ambient temperature, initial deformation, or the current that passed through it. It was observed that there was a certain repeatability in the tests if the initial stress and deformation were preserved. If this were not true, for equal values of PWM, the force exerted by the SMA differed significantly. Minor variations in ambient temperature (25–27 °C) seemed not to have had much influence. Therefore, the strength exerted by the SMA depended both on the current that circulated and the initial deformation. It made open-chain control practically impossible and complicated it in a closed chain by introducing this new variable to consider.

**Figure 11:**
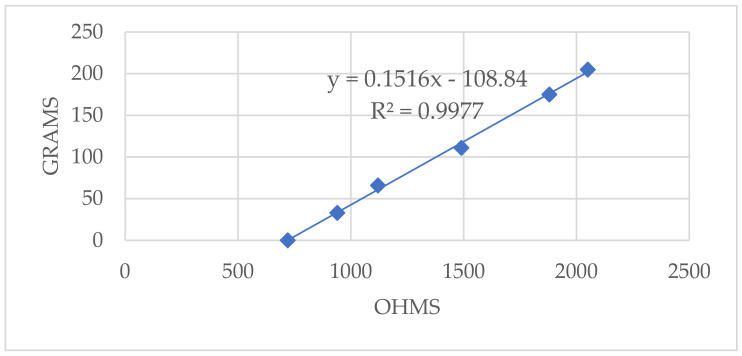
Results of the sensor characterization tests for the elaboration of the Resistance–Weight function with the equation of the line and correlation coefficient.

Then the elastic sensor was characterized to perform a closed-loop control of the SMA. For this, the initial assembly was used by putting the sensor in place of the SMA. Then, with the help of a multimeter, the resistance in the sensor was measured. The data obtained can be seen in Figure 11.

There was a clear relationship between strength and resistance where the points fit perfectly (correlation coefficient of 0.9977) to a line. The slope of the line was m = 0.1516. The ordinate at the origin was equal to *n* = −108.84. The equation that therefore related the voltage in the SMA-Sensor assembly as indicated in Equation (2) was

(2) $$T=\frac{(0.1516\;\left(\frac{V_{in}\;R_{gauge}}{V_{A0}}-R_{gauge}\right)-108.84}{9.81}\;\left[N\right],$$

Gauge resistors were used to read the sensors to obtain an average of their resistance using a voltage divider and, consequently, determine the relationship between the electrical resistance and the force experienced. Therefore, the tension state of the system was defined.

The regulator designed to adjust the PWM to the requested strength worked on the error between the demanded force and the desired one. It worked in grams as the unit of measurement of the error for convenience and facilitated the calculations to the Arduino.

After numerous tests at room temperature (25–27 °C), we concluded that the value 7 of the PWM provided good thermal stability to the system for the range of forces (0–2 N) and the initial deformation of the spring with which it will work.

Therefore, as long as were within ±1 g, this was the PWM exerted. If we were between +1 and +2, +2 and +5, or higher than +8, it was 6, 5, and 4, respectively. If we were between −2 and −1, −5 and −2, or −5 and −8, it was 8, 9, and 10, respectively. If it was less than −8, the SMA went directly to the warm-up phase.

**Figure 12:**
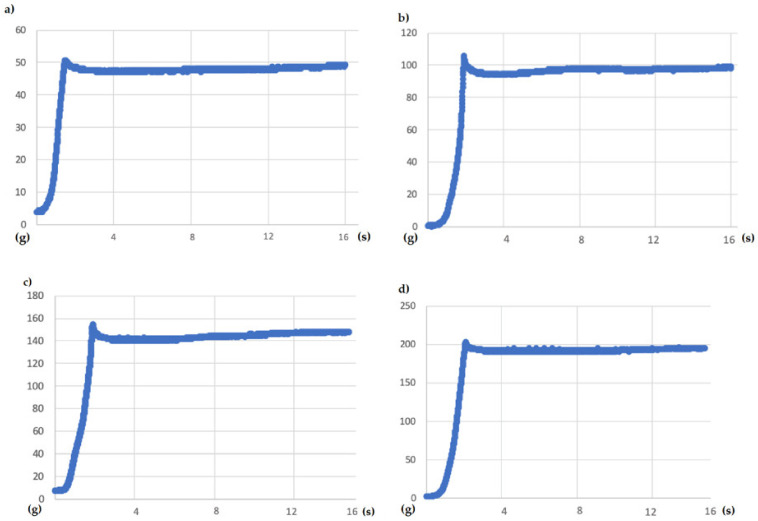
Weight vs. time graph for a 15 s test, and a demanded weight of (**a**) 50 g (**b**) 100 g (**c**) 150 g (**d**) 200 g.

The tests carried out had a duration of 15 s. Therefore, first, forces of 50, 100, 150, and finally 200 g were requested. The results are represented in the following graphs in Figure 12.

The existing initial peaks did not exceed 8 g of difference with the requested force, and the falls that followed them were not far from more than 8 g in any case because there would be peaks in the graphs due to entering the heating phase. Then it managed to stabilize at the indicated value. Therefore, we affirmed that it was a pretty precise system, and it allowed us to exercise reliable, albeit slow, control.

**Figure 13:**
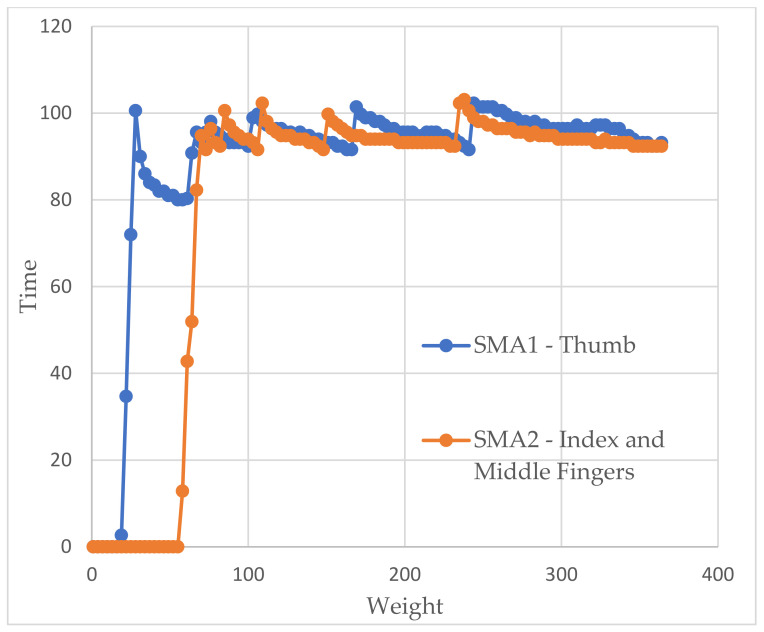
Weight vs time graph for the glove test with a simulated weight of 100 g.

**Figure 14:**
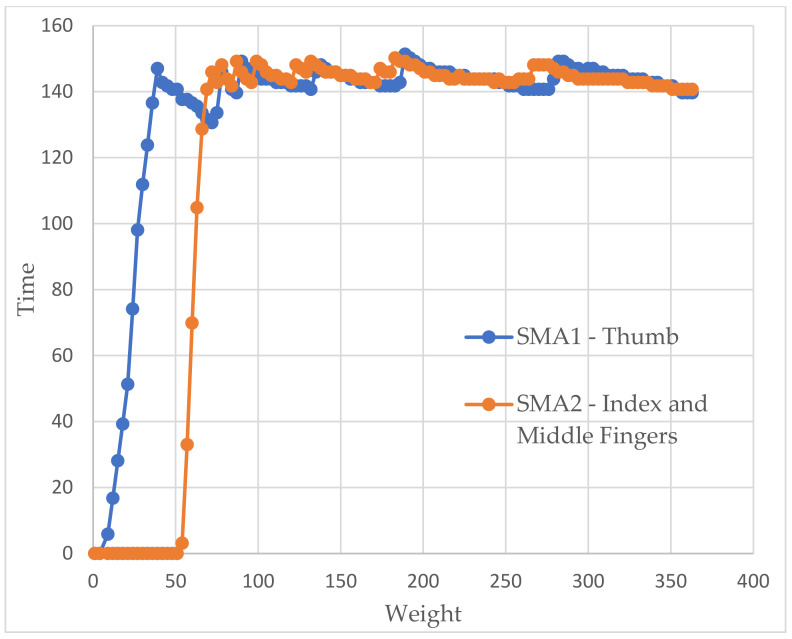
Weight vs time graph for the glove test with a simulated weight of 150 g.

In the glove tests (showed in Figure 13 and Figure 14), as we did in the previous ones, we tried to obtain weight–time graphs, with the glove already mounted on the arm, to see if they corresponded to those already achieved. Each SMA is represented in a different color to illustrate the operation of both. The first thing that struck us was the delay between them, which corresponded to approximately 2 s. By not being able to heat both together, there was no choice but to accept this behavior.

We also see how the force in the first SMA decays as the second begins to heat up since no energy is being supplied. Thus, all of it has to go to the second dock. However, once the latter has warmed up, the former recovers quickly.

As before, we saw how the force tended to stabilize a little below the desired values with slightly higher errors but still lower than 10%. However, a behavior appeared that we did not observe before. These were small peaks of force. The system detected that the springs had to be heated again because the error was in the last available window (8 g). If we looked closely, when one of these warm-ups ended, the other one immediately began.

Factors such as the person’s pulse or being faced with a new sensation could have cause strange movements that ended up resulting in a loss of balance point that, finally, lead to those peaks. They did not appear before as they were in a completely static system.

## 5. Tests

The glove was tested with 15 participants to determine if it could convey a haptic feeling of grip. Volunteers aged between 16 and 56 years (10 females and 5 males) had no previous experience with this type of device. Consequently, training was carried out. Different forces were simulated with the glove, while on the other hand, the participants held an object of the same simulated weight so that they could notice the type of sensations to be experienced.

After the training, they experimented with the forces (in random order) with eyes closed. The device allowed the simulation of three different forces: 100, 200 and 300 g. They had to determine which one was being simulated at any given moment. Four different types of tests were presented. In each one, the sequence of simulated forces varied. In the first test, they were simulated in increasing order (100–200–300), in the second, decreasing (300–200–100), and in the third and fourth, there was no logical order (200–100–300 and 200–300–100, respectively).

At the end of the tests, the participants had to answer two blocks of questions. In the first, the questions were general, and the results are presented in Table 3. The diagram below Table 3 summarizes the results of the questions. It shows the mean between questions about the same topic (for example, between questions 1 and 5) to calculate a single value for the essential characteristics of the glove. The participants assigned a value from 0 to 10 to each question. Zero corresponded to total disagreement with the proposed proposition and, 10 corresponds to complete agreement.

**Table 3:** Results of questions about general aspects in a scale of 0 to 10 (where 0 = worst, 10 = best).

Questions	Avg.	σ
(1) I felt comfortable wearing the device.	7.3	1.94
(2) The device is lightweight.	8.3	1.83
(3) The device was easy to put on and take off.	7.0	1.41
(4) The device was easy to use.	8.6	1.06
(5) The device did not cause any kind of discomfort (e.g., heat, weight).	8.8	2.08
(6) The device performed the function it promised.	7.8	1.32
(7) I was able to clearly appreciate the different forces the device performs.	9.0	1.46
(8) I was satisfied with the overall experience.	8.3	1.23
(9) Previous training was helpful.	8.0	1.06
(10) Sensitivity improved with the use of the device.	9.5	0.64


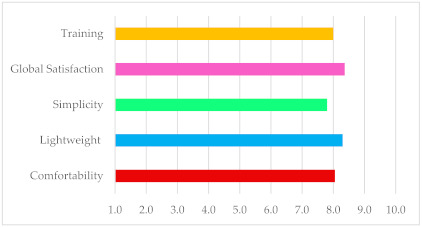


In the second block, the answers during the test were annotated. The absolute error is showed in Table 4. The diagram below Table 4 graphically describes the results of the tests. Each color corresponds to a specific weight, and the percentage of errors is shown.

**Table 4:** Results of tests.

Test	Simulation Order (g)	Error	Avg. Error
Test 1	100	26.67%	20%
	200	33.33%	
	300	0.00%	
Test 2	300	20.00%	13.33%
	200	20.00%	
	100	0.00%	
Test 3	200	13.33%	11.11%
	100	0.00%	
	300	20.00%	
Test 4	200	13.33%	4.44%
	300	0.00%	
	100	0.00%	


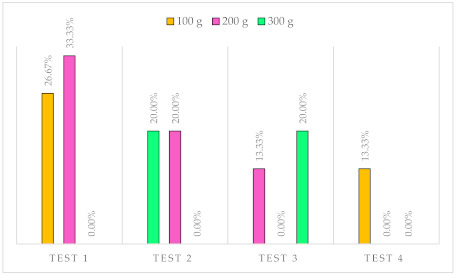


The percentage of success in the tests was 83.7%. It would improve considerably if more tests were carried out since, as we saw, results improve markedly with use, going from an error of 20% in the first test to 4.44% in the last. Most mistakes were made by confusing the force of 200 with that of 300. Specifically, the simulation of 300 g had a correctness of 86.7% and that of 200, 73.3%. Many of these errors ended up being recognized by the participants.

This result was satisfactory since even with objects, it was not easy to distinguish two forces of this type if they were not taken consecutively. The percentage of success with 100 g amounted to 91.1%. It was an expected result: the relative jump from 100 to 200 was 100%, and the one between 200 and 300, 50%. In addition, the participants perfectly distinguished between a minimum level of action (100 g) and no actuation.

Considering the questions asked, we concluded that the level of satisfaction was high, especially in questions regarding the development of the tests. Comfort was the second most penalized factor in voting. Because the participants had short forearms, the bracelet had to be placed above the elbow for some tests because, as we said before, there had to be a minimum length in the spring–sensor assembly to ensure correct operation. It created some discomfort.

## 6. Conclusions

This paper showed the development of a soft haptic glove that allows simulating small forces. The SMA springs were used as actuators because of their excellent power-to-weight ratio. In addition, the device is soft, lightweight, and easy to put on.

The glove allowed the simulation of three different forces (100, 200 and 300 g) and generated in the user the sensation of holding an object. To re-feed the glove, elastic sensors were used. Thus, they had a double function: on the one hand, they helped the springs to recover in the cooling phase, and on the other they provide us with information about the force being exerted. In addition, several experiments were carried out to characterize the springs used in the prototype to carry out correct control of them.

Tests with real users were carried out to validate the device. In the first phase, users with the glove on experienced different forces in causal order. People guessed the force they were experiencing with an initial precision of 80% and a final one of 95.56%. It showed that after a few uses, people recognized without problems the sensation they were experiencing. Users were also asked about their experience with the glove to determine the level of comfort. The answers showed a high level of satisfaction with the device and an overall good experience.

In conclusion, the SMA springs combined with elastic sensors allowed more precise control of this type of actuator. Nevertheless, the device needs some improvement in the design. For example, it will be interesting to add a cooling system to speed up the cooling process to allow users to grip different objects faster. Another interesting future work would be to develop an application with virtual reality.

## Figures and Tables

**Table 1:** Nitinol springs’ characteristics.

Nitinol Springs Dimensions	
Total length including eyelets	25 mm
Outer diameter	6 mm
Cable diameter	0.75 mm
Number of coils	21
Activation temperature	65 °C

**Table 2:** Material parameters used for simulation of SMA helical spring.

Material Properties for A Spring Actuator	Values
Density (kg/m^3^)	4650
Elastic modulus for austenite phase (MPa)	51,700
Elastic modulus for martensite phase (MPa)	51,700
Poisson’s ratio	0.33
H (Hardening Parameter) (MPa)	1000
R (Elastic Limit) (MPa)	140
B (Temperature Scaling Parameter) (MPa K^−1^)	5.6
T_0_ (Reference Temperature) (K)	250
M	0
Maximum Transformation Strain (mm mm^−1^)	0.04

## Data Availability

Not applicable.
